# Supplementary material for: Protein Biomarkers Enable Sensitive and Specific Cervical Intraepithelial Neoplasia (CIN) II/III+ Detection: One Step Closer to Universal Cervical Cancer Screening
Source: Cancers (Basel). 2025 May 24;17(11):1763. doi: 10.3390/cancers17111763 (PMC12153835; doi:10.3390/cancers17111763)
Supplement: Supplementary file 1 [file cancers-17-01763-s001.zip › cancers-3633734-supplementary.pdf]

Supplementary Materials

Protein Biomarkers Enable Sensitive and Specific Cervical Intraepithelial Neoplasia (CIN) II/III+ Detection: One Step Closer to Universal Cervical Cancer Screening

Samrin F. Habbani [1,†], Sayeh Dowlatshahi [2,†], Nathanael Lichti [3], Meaghan Broman [1], Lucy Tecle [2], Scott Bolton [2], Lisa Flowers [4], Rafael Guerrero-Preston [5], Jacqueline C. Linnes [2] and Sulma I. Mohammed [1,6,*]

[1] Department of Comparative Pathobiology, Purdue University, West Lafayette, IN, USA

[2] Weldon School of Biomedical Engineering, Purdue University, West Lafayette, IN, USA

[3] Bindley Bioscience Center, Purdue University, West Lafayette, IN, USA

[4] Emory University School of Medicine, Atlanta, GA, USA

[5] LifeGene Biomarks, Toa Baja, Puerto Rico

[6] Department of Small Animal Clinical Sciences, Cancer Control and Population Sciences, University of Florida Health Cancer Center, Gainesville, FL, USA

[*] Correspondence: Sulma I. Mohammed, e-mail: smohammed2@ufl.edu

[†] These authors contributed equally to this work.

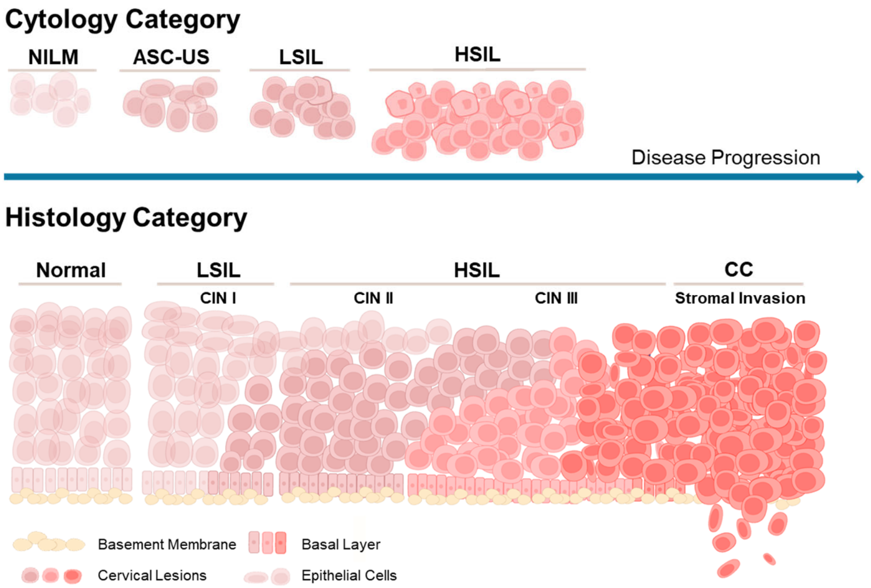

Figure S1. Schematic representation of cervical disease progression and its different cytology and histology categories. (NILM: Negative for intraepithelial lesion or malignancy, ASC-US: Atypical squamous cells of undetermined significance, LSIL: Low-grade intraepithelial lesion, HSIL: High-grade intraepithelial lesion, CIN: Cervical intraepithelial neoplasia, CC: Cervical cancer.)

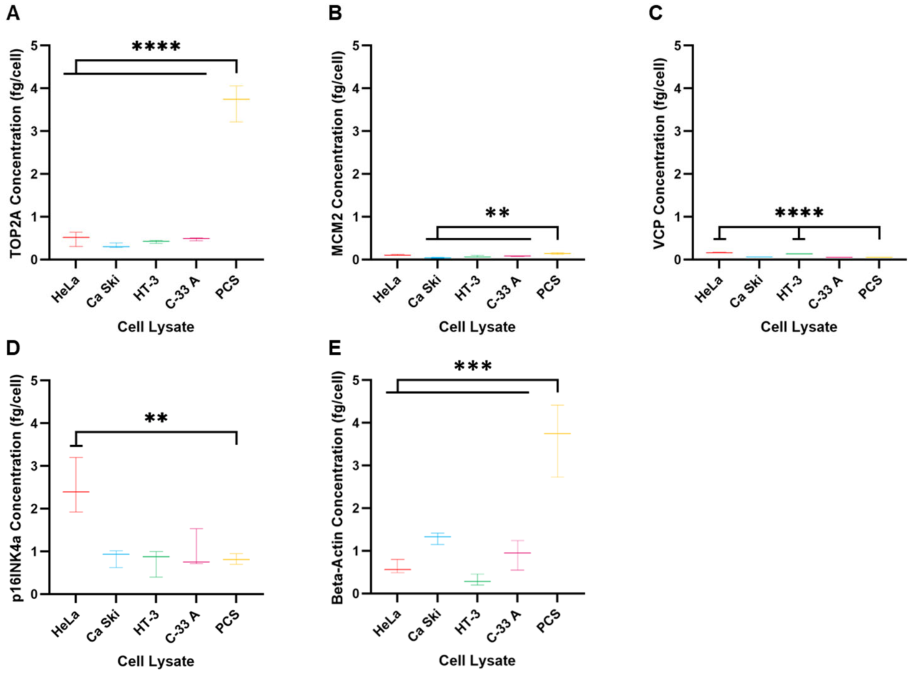

Figure S2. Biomarker expression reported as protein amount per cell in lysates of cervical cancer cell lines and PCS cells. ELISA measurements for (**A**) TOP2A, (**B**) MCM2, (**C**) VCP, (**D**) p16INK4a, and (**E**) beta-actin in lysates of HeLa, Ca Ski, HT-3, and C-33 A cancer cell lines, and PCS cells. (Dunnett's multiple comparisons test: **: $p < 0.01$, ***: $p < 0.001$, ****: $p < 0.0001$, $n = 3$. Boxplots represent the median (bar) and range (whiskers) of the data.)

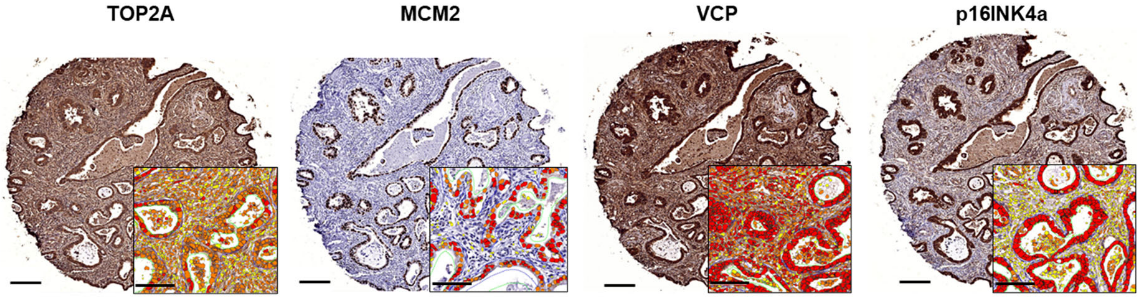

Figure S3. Immunostaining of biomarkers in cervical ADC tissues. Representative original IHC-stained sections demonstrating TOP2A, MCM2, VCP, and p16INK4a expression in cervical ADC tissues. The computationally pseudocolor-coded images (insets) were quantified using the Visiopharm software, version 2024.07.2.17285x64. Brown represents the original IHC staining and purple denotes the nuclear counterstain (scale bars = 200 μm), while pseudocolors indicate staining intensity: red (strong), orange (moderate), and yellow (weak) (scale bars = 100 μm).
